# Prevalence of Computed Tomography Overuse for Mild Head Injury in Adults

**DOI:** 10.7759/cureus.35551

**Published:** 2023-02-27

**Authors:** Bedoor Al Omran, Jayaditya Devpal Patil, Alekya Anala, Prianna Menezes, Noora Ahmed, Iman Cheffi, Salah Alghanem

**Affiliations:** 1 Department of Radiology, Bahrain Defence Force Hospital, Riffa, BHR; 2 School of Medicine, Royal College of Surgeons in Ireland-Medical University of Bahrain, Busaiteen, BHR; 3 Department of Surgery, Bahrain Defence Force Hospital, Riffa, BHR; 4 Department of Emergency Medicine, Bahrain Defence Force Hospital, Riffa, BHR

**Keywords:** overuse of ct, mild head injury, trauma imaging, canadian ct head rule, computed tomography (ct )

## Abstract

Introduction: The Canadian CT Head Rule (CCHR) is one of many established guidelines for assessing the need for computed tomography (CT) imaging in patients with minor head injuries. Adhering to such criteria would promote the appropriate use of CT imaging, lower healthcare expenses, and prevent harmful radiation exposure. There is no current literature assessing the overuse of CT imaging for minor head injuries in the Kingdom of Bahrain. This study aims to evaluate CT overuse in adult patients with minor head trauma.

Methods: The study was conducted at the Bahrain Defense Force Hospital over 12 months from January to December 2021. All adult patients (>14 years) who sustained a minor head injury and were referred to the emergency department for CT brain imaging were included in the study. Patients presenting for other reasons or suffering from moderate to severe head injuries were excluded. CT reports were retrieved for analysis. The CCHR was used as a reference.

Results: A total of 486 CT scans were performed. Loss of consciousness was the most common symptom on presentation (n = 74 cases). Only 12.1% of CT scans reported positive findings. The prevalence of CT overuse was highest in patients aged 21-30 years. Patients presenting with loss of consciousness showed a high overuse of CT imaging, accounting for 20.3% of all cases. Only 77.4% of cases met the CCHR criteria and 22.6% were defined as overuse, with 95% confidence interval (0.189, 0.266).

Conclusion: When referring to the CCHR, CT imaging for a minor head injury in adults was overused in 22.6% of cases. Further research will be required to reveal the underlying reasons for these findings along with interventions to reduce future overuse.

## Introduction

Computed tomography (CT) scan of the head has long been the imaging modality of choice for rapid and reliable diagnosis of neurocranial pathologies such as skull fractures, epidural hematoma, and contusions that have occurred secondary to trauma. Using CT for examining patients with minor head injuries can be safely limited to those with specific clinical signs. Some clinical and patient risk indicators, such as age, consciousness impairment, focal neurological deficit, and time interval between head trauma and CT imaging, can be used as a source of valuable information for further investigation [1-3]. Several national and international guidelines for CT imaging in patients with mild head injuries have been published; some of these guidelines are based in part on published algorithms, such as the New Orleans Criterion (NOC) and the Canadian CT Head Rule (CCHR) [4-7]. The implementation of the CCHR was associated with a slight reduction in CT utilization and a higher diagnostic yield of head CTs for adult trauma contacts in community emergency departments [8]. Implementing such criteria has the critical purpose of only performing CT scans on appropriate patients who are at risk of developing complications. This approach would lower healthcare expenses, relieve workload, and avoid unnecessary radiation exposure [9-11]. Often, the final decision to proceed with CT imaging rests with the physician, who can screen all medium- and high-risk patients (lenient criteria) or scan only high-risk patients (strict criteria) [12]. There is much overlap between published guidelines and significant variances in terms of risk factor definitions. Some recommendations suggest a more liberal approach to CT use, while others urge a more stringent one [7,13]. CT scanning is not recommended for low-risk patients; however, its overuse has plagued the healthcare sector globally. Some studies monitoring the overuse of CT imaging have shown that 10-35% of scans performed for minor brain injury were not recommended, while other authors endorse 20-50% of imaging performed to be unnecessary [12,14]. A very common presentation to the emergency department is a fall in elderly patients, where studies have found the systematic indication of an emergency CT scan of the brain to have low diagnostic and therapeutic yield [2]. CT scans for patients presenting with vertigo have also been shown to yield poor results, with significant findings noted in only 6.9% of cases [15]. Today, a majority of healthcare professionals examine diverse techniques to prevent overutilization of CT imaging [12,16]. Currently, there is no literature studying the overuse of CT imaging in cases of minor head trauma in the Kingdom of Bahrain. This study aims to highlight the extent of CT overuse in adult patients with minor head trauma. The results from this study will allow for a better understanding of guideline compliance and aid in potentially establishing and enforcing updated management algorithms. 

## Materials and methods

This retrospective study was conducted at the Bahrain Defense Force-Royal Military Services (BDF-RMS) hospital over a 12-month period from January 2021 to December 2021. All adult patients, defined as patients older than 14 years, who sustained a minor head injury, defined as loss of consciousness, definite amnesia, or witnessed disorientation with a Glasgow Coma Scale (GCS) of 13-15, and who were referred from the emergency department for a CT head scan were included in our study [14]. Adult patients presenting to the emergency department for any other reason or patients suffering from moderate to severe head injuries were excluded. All radiological reports were retrieved from the electronic database to analyze for positive findings. The CCHR was used as a reference to assess the extent of CT brain overuse at our institute. This study was approved by the BDF-RMS Research and Research Ethics Committee. 

## Results

A total of 486 CT scans were performed for minor head injuries and were included in our data analysis, of which 68.1% (n = 331) were males and 31.9% (n = 155) were females. The cohort’s age ranged from 14 to 101 years, with a mean age and standard deviation of 44.86 ± 21.609. The demographics and patient characteristics are summarized in Table 1. 

**Table 1:** Demographics and patient’s characteristics frequencies. Some patients had multi-injuries, symptoms, and radiological findings. n: number of patients; LOC: loss of consciousness; CT: computed tomography.

	n (%)
Age (years)	
≤20	58 (11.9)
21–30	105 (21.6)
31–40	86 (17.7)
41–50	57 (11.7)
51–60	38 (7.8)
61–70	59 (12.1)
71–80	51 (10.5)
>80	32 (6.6)
Gender	
Male	331 (68.1)
Female	155 (31.9)
Injury	
Fall	188 (38.7)
Motor vehicle accidents	157 (32.3)
Witness	68 (14)
Symptoms	
LOC	74 (15.2)
Vomiting	20 (4.1)
Seizure	9 (1.9)
Scalp wound	19 (3.9)
Positive CT findings	
Subdural hematoma	10 (2.1)
Epidural hematoma	3 (0.6)
Intracranial hemorrhage	6 (1.2)
Skull fracture	9 (1.9)
Edema	5 (1.0)
Axon injury	3 (0.6)
Sub arachnoid hemorrhage	11 (2.3)
Ventricular hemorrhage	2 (0.4)
Infract	4 (0.8)
Lesion	4 (0.8)
Scalp hematoma	2 (0.4)

**Figure 1:**
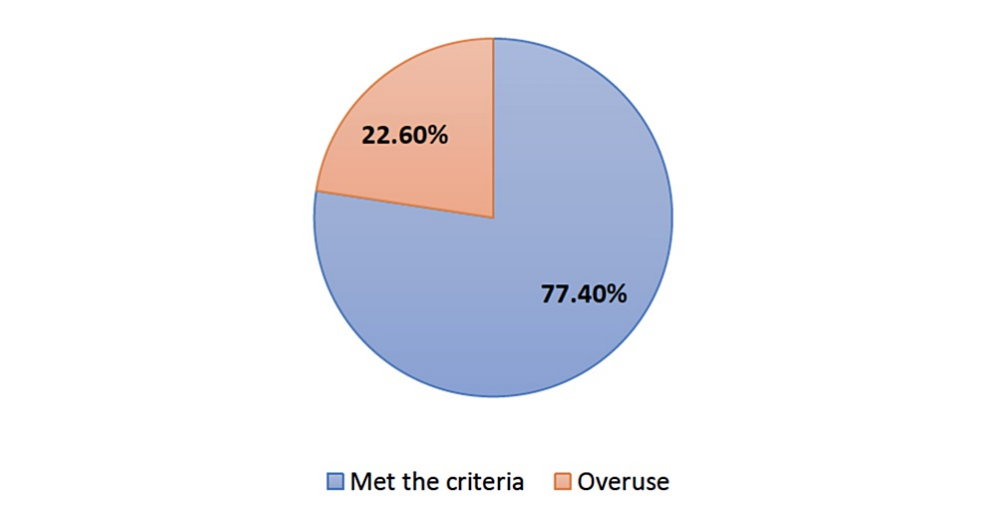
Overall percentages of CT scanning overuse.

The most common symptom on presentation was loss of consciousness (LOC) with 74 cases. Fifty-nine (12.1%) positive CT scan findings were reported, with subarachnoid hemorrhage and subdural hematoma having the most prevalence. Patients aged between 21 and 30 showed the highest prevalence of CT scan overuse, with 28 (25.5%) cases. Overall percentages of CT scanning overuse are represented in Figure 1.

**Figure 2:**
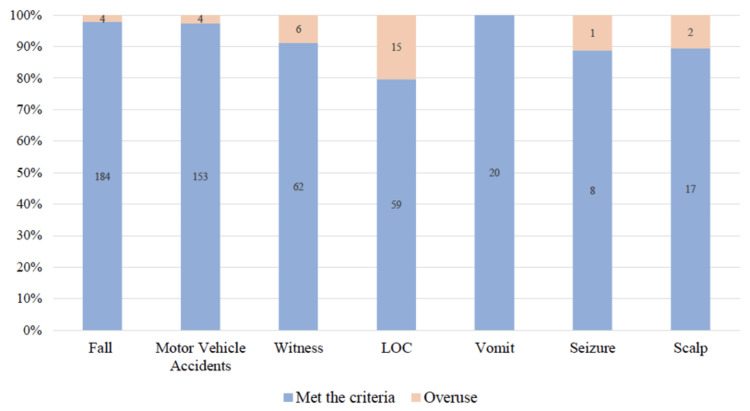
Frequencies of injuries and symptoms in meeting CT scan criteria. LOC: loss of consciousness.

Of the 486 CT scan cases, 376 (77.4%) cases met the CCHR criteria, and 110 (22.6%) were defined as overuse, with 95% confidence interval (0.189, 0.266). The frequency of injuries and symptoms in meeting CT scan criteria are illustrated in Figure 2. Patients presenting with LOC showed high overuse of CT imaging in 15 (20.3%) cases, as highlighted in Figure 2. 

## Discussion

CT head imaging is an essential tool in diagnosing post-traumatic cranial injuries. They have also been suggested to detect neurological pathology due to minor head trauma that would require immediate intervention. Due to the potential fear of misdiagnosing or completely missing fatal findings, CT imaging has been vastly overused. Such practice can result in unnecessary exposure to radiation, increase the risk of cancer, and financially burden the healthcare sector [17]. The utility of routine CT scanning in patients with mild head injury is debatable and controversial for both young and adult patients. To determine the need for CT imaging, various clinical guidelines have been developed. In this study, we applied the CCHR and observed the overuse of CT scan imaging in 110 cases (22.6%). Multiple studies in the current literature have reported similar findings with varying degrees of overuse when adhering to the CCHR. Two studies that utilized the CCHR reported a higher percentage of CT overuse compared to our study. The paper by Shobeirian et al. in Iran, assessing 170 adult patients with mild head trauma, reported 31.8% of CT head imaging overuse when referring to the CCHR [18]. Another analysis of CT overuse in minor traumatic brain injury by Melnick et al., assessing 346 adult patients, reported an even higher percentage (35%) of CT overuse [14]. A study by Klang et al. that followed the CCHR reported a lower percentage (10.9%) of overuse in the general population but 37.3% overuse when analyzing patients younger than 65 years [19]. These findings indicate a potential relationship between the extent of overuse and patient age. The highest reported findings were seen in a study by Cellina et al. with 72% overuse [20]. The discrepancies in the extent of CT overuse across the studies may be attributed to factors such as variability in clinical settings, resource availability, and local guidelines. The BDF-RMS offers accessible imaging modalities that could potentially play a role in the practice of imaging overuse. Interestingly, our study found young patients aged between 21 and 30 to have the highest overuse of CT head imaging, with the second highest in patients between 31 and 40. Data from the study by Shobeirian et al. correlate with our findings in this aspect; they reported 34% of overuse in young patients compared to 20.3% in our study [18]. Another study by Cellina et al., conducted exclusively on patients aged 18-45, evaluating a total of 493 CT scans, observed an overuse of 72%. This overuse in the population may be attributed to the referring physician and the mechanism of trauma involved [20,21]. Collectively, these results emphasizes the importance of reducing the overuse of CT head imaging, especially in young adults, where early exposure to radiation may allow more time for the establishment of malignant neoplastic potential. From our results, patients who presented with LOC also had a higher overuse of CT imaging. A study conducted in a trauma center in the USA corroborates these findings. Their research reported that only 8.1% of patients presenting with LOC had significant CT findings [21]. The four major and commonly utilized criteria include the National Institute for Health and Clinical Excellence (NICE), the NOC, the American College of Emergency Physicians (ACEP), and the CCHR. Overall, there is no consensus about one criterion being superior; however, according to a meta-analysis of economic evaluations, the CCHR was found to be the most cost-effective option in imaging mild head trauma patients [22]. It is also one of the most utilized protocols in the emergency department for traumatic brain injury, making it an ideal selection for our study [19]. An observational study that assessed the pre- and post-intervention of the CCHR in 13 Southern California community emergency departments found a 5% reduction in CT head imaging post-intervention [8]. Another study also claimed approximately 37.3% of trauma CT head scans could be avoided if guidelines were followed [23]. This has been supported in a study by Adam et al., demonstrating a potential 36.8% reduction of CT head imaging if the CCHR was adhered to [11]. Although our study produced significant results, data were retrieved from a single medical institute. To reduce bias and increase reliability, further training is required for emergency physicians to adhere to the regulations and further communication with the radiologists to enhance the imaging results. A prospective study bearing these results in mind may allow for future studies to improve overall patient management and outcomes.

## Conclusions

When referring to the CCHR, CT brain imaging for minor head injuries in adults was overused in 22.6% of patients in our study. The prevalence of CT overuse was highest in patients aged 21-30 years and only 12.1% of CT scans performed reported positive findings. This study provides valuable data on the extent of overuse of CT brain imaging and encourages further studies in the field, which will allow for a better understanding of this phenomenon and its impact on patient management. A national multi-center study would provide a more comprehensive picture of the overall pattern and distribution of CT overuse in the Kingdom of Bahrain. 
